# Occupational class differences in outcomes after ischemic stroke: a prospective observational study

**DOI:** 10.1186/s12889-021-11624-9

**Published:** 2021-08-19

**Authors:** Yinwei Zhu, Yaling Lu, Mo Zhou, Ping Huang, Peipei Zhang, Yumei Guo, Liying Lv, Yonghong Zhang, Chongke Zhong, Tan Xu

**Affiliations:** 1 Department of Epidemiology, School of Public Health and Jiangsu Key Laboratory of Preventive and Translational Medicine for Geriatric Diseases, Medical College of Soochow University, 199 Renai Road, Industrial Park District, Suzhou, Jiangsu Province, 215123 People’s Republic of China; 2 Department of Science and Research, The Affiliated Suzhou hospital of Nanjing Medical University, Suzhou, Jiangsu People’s Republic of China; 3 Medical Records Room, Changzhou Traditional Chinese Medicine Hospital, Changzhou, Jiangsu People’s Republic of China; 4 Department of Acute Infectious Disease Control and Prevention, Changzhou Center for Disease Control and Prevention, Changzhou, Jiangsu People’s Republic of China; 5 Clinical Laboratory, Inner Mongolia Xingan League People’s Hospital, Xingan League, Inner Mongolia, People’s Republic of China

**Keywords:** Occupation, Ischemic stroke, Cardiovascular events, White-collar worker, Farmer, Prognosis

## Abstract

**Background:**

Occupational class is an integral part of socioeconomic status. The studies focused on the occupational difference in ischemic stroke outcome in a Chinese population are limited. We aimed to investigate the associations between occupational class and the prognosis of patients with ischemic stroke in China.

**Methods:**

We included 1484 ischemic stroke participants (mean age: 63.42 ± 11.26 years) from the prospective cohort study: Infectious Factors, Inflammatory Markers and Prognosis of Acute Ischemic Stroke (IIPAIS). Occupational class was categorized into white-collar workers, blue-collar workers and farmers in our study. Study outcomes were cardiovascular events and all-cause mortality within 12 months after ischemic stroke onset. We applied Cox proportional hazard model to evaluate the associations between the occupational class and study outcomes after ischemic stroke.

**Results:**

Within 12 months after ischemic stroke, there were 106 (7.5%) cardiovascular events and 69 (4.9%) all-cause deaths. The Kaplan–Meier plots showed that white-collar workers had highest risk of cardiovascular events after 12-month follow-up (Log-rank *P* = 0.02). Multivariate adjusted hazard ratio and 95% confidence intervals (CIs) of farmers versus white-collar workers was 0.43(0.20–0.91) for cardiovascular events. No significant difference showed in blue-collar workers versus white-collar workers, with fully adjusted hazard ratio 0.62(95% CIs, 0.23–1.67).

**Conclusions:**

Compared with white-collar workers, farmers are associated with less risk of cardiovascular events at 12 months after ischemic stroke, while there are no significant differences in blue-collar workers.

## Background

Stroke is the second leading cause of disability adjusted life years in the old around the world [1]. According to the data from National Epidemiological Survey of Stroke in China 2013, the age-specific prevalence of stroke increased with age, especially in those aged ≥50 years [2]. In China, individuals with ischemic stroke have a relatively poor prognosis after first stroke, with major vascular event rate of 27% at 1 year and 45% at 5 years [3]. China is facing severe challenges to the disease burden from stroke. Socioeconomic status, typically including income, education, employment and social status, is widely believed to have link with the incidence and outcomes of the stroke [4, 5]. Pierre Amarenco et found that low levels of socioeconomic status are associated with vascular events and ischemic stroke [6, 7]. Making sense of the influence of socioeconomic factors on outcomes of stroke has valuable public health significance for improving stroke prognosis.

The occupation often means the work in which individuals serve the community and serve as a major source of livelihood. The occupational experiences have profound effects on physical and mental health by some means. On the one hand, a job can offer both intellectually and socially enriched environment, supporting cognitive function. On the other hand, job is one of the main sources of stress among employees. Emerging studies had revealed that various kinds of stress at work have been related to poorer cognitive ability [8, 9].

As a well-known indicator of socioeconomic status, occupational class is considered to be a key factor of the development of cardiovascular diseases. There are varying backgrounds, prospects and outcomes in different occupations, and these are not given across or even within occupations [10]. Thus, there exists health difference among various occupational populations. For instance, those with low occupational status often have high prevalence of risk factors, such as low quality of care and limited access to high-quality medical resource [11]. Previous studies reported that white collars have more risk suffering from poor prognostic factors of stroke, like diabetes and hypertension [12, 13]. However, the findings on the associations between occupational status and stroke prognosis are not consistent. Several findings reported that low occupation increased risk of stroke mortality [14, 15], while others showed no significant associations [16, 17].

Knowledge of disparities in outcomes after stroke is of great importance for effective stroke management and prognosis improvement. Yet, occupation status differs in clinical and lifestyle factors and many of the existing studies were aimed to examine occupational disparities in single outcome of stroke [15, 18]. Moreover, the studies focused on the associations of occupation class with outcomes of stroke in Chinese population were scarce. Therefore, we investigated the association between occupational class and cardiovascular events and all-cause mortality after stroke onset among white-collar, blue-collar workers and farmers in China.

## Materials and methods

### Study design and population

The multicenter prospective cohort study Infectious Factors, Inflammatory Markers and Prognosis of Acute Ischemic Stroke (IIPAIS) was designed to evaluate the associations of infectious factors and inflammatory markers with clinical outcomes of ischemic stroke [19]. It recruited a total of 1711 patients aged ≥40 years with first-ever ischemic stroke from June 2011 to December 2013 in 25 hospitals of five provinces (Jilin, Liaoning, Jiangsu, Hebei and Henan) and two autonomous regions (Inner Mongolia and Ningxia) across China. All included ischemic stroke patients were confirmed by computed tomography or magnetic resonance imaging of the brain within 72 h of symptom onset. Furthermore, the IIPAIS excluded patients with subarachnoid hemorrhage, transient ischemic attack, cerebral hemorrhage, or hemorrhage caused by tumor or hematologic diseases, along with pregnant women. The present study was a secondary analysis of IIPAIS, which was designed to find the associations between occupation class and ischemic stroke prognosis. For the present study, a total of 1484 ischemic patients were included, excluding 40 patients (2.3%) who lost 1-year follow-up and 187 patients whose occupation information could not accord with the inclusion criteria of the study.

IIPAIS was approved by the institutional review boards at Tulane University in the United States and Soochow University in China. All patients engaged in the research provided written informed consents.

### Data collection

We used a standard questionnaire to collect baseline data with respect to demographic characteristics, medical history and clinical features at the time of enrollment. Trained neurologists applied the National Institutes of Health Stroke Scale (NIHSS) at baseline to assess the stroke severity. Trial of Org 10,172 in Acute Stroke Treatment (TOAST) criteria was used to classify the ischemic stroke subtypes as large-artery atherosclerosis (thrombotic), cardiac embolism (embolic), small-vessel occlusion (lacunar), stroke of other determined etiologies, and stroke of undetermined etiology, according to the symptoms and imaging data of the patients by experienced neurologists [20]. The blood pressure (BP) was measured by trained nurses when the patient was in the supine position, according to a standard protocol adapted from procedures recommended by the American Heart Association [21]. At admission, all participants took routine serologic tests (fasting plasma glucose, blood lipids, creatinine, etc.) in corresponding participating hospital.

### Occupation class

Study participants self-reported their longest-held occupations, whose classification was based on Labor Law of the People’s Republic of China. According to the existing researches [22, 23], we classified occupation into three groups: white-collar workers including office workers, managers and professional technician; blue-collar workers including technicians and machine operators; and farmers. Homemakers and freelance were excluded because of limited quantity.

### Assessment of outcomes

Participants were followed up in person for 12 months after onset or hospital discharge by trained neurologists who were unaware of treatment assignment. In the analysis, the outcomes were defined as cardiovascular events and all-cause mortality. Cardiovascular events included recurrent stroke, myocardial infarction, heart failure, pulmonary embolism and peripheral arterial disease, which were abstracted from related hospital. The causes and date of death were verified by examining hospital medical records. The outcome assessment committee reviewed and adjudicated vascular events based on the criteria established in the Antihypertensive and Lipid-Lowering Treatment to Prevent Heart Attack Trial (ALLHAT) [19].

### Statistical analysis

All participants were divided into 3 subgroups according to self-reported occupation. Basic characteristics of the study population were described by means with standard deviation (SD), median with interquartile range (IQR) or frequencies with percentages, which were compared between the 3 groups, using the variance analysis or the χ^2^ test, as appropriate.

We calculated the incidence density to describe the study outcomes in different groups. The Kaplan-Meier curve was applied to compare the different prognosis among the groups with log-rank test. To further investigate the association between occupation and ischemic stroke prognosis, Cox proportional hazard model was used to calculate hazard ratios (HRs) and 95% confidence intervals (CIs) for the associations of occupational class with the stroke prognosis. The white-collar group served as the reference group for the analyses. We performed 3 Cox proportional hazard models. Model 1 was an unadjusted model. Model 2 only adjusted for age, sex. Model 3 included the factors in model 2 as well as current cigarette smoking, current alcohol drinking, time from stroke onset to hospitalization, ischemic stroke subtype, baseline NIHSS score, diastolic blood pressure, high-density lipoprotein cholesterol, fasting plasma glucose, medical history (hypertension, hyperlipidemia, coronary heart disease, diabetes mellitus, family history of stroke), treatment during hospitalization (hypoglycemic, anticoagulants, antiplatelet agents, thrombolysis), NIHSS score at discharge, education, urban and employed.

In addition, subgroup analysis was performed to test whether the associations between occupational class (white-collars and farmers) and ischemic stroke prognosis was modified by age, sex, education, race, urban, current cigarette smoking, current alcohol drinking, baseline NIHSS score and history of hypertension. Multiple imputation for missing data was performed using the Markov chain Monten Carlo method. All *P* values were two tailed, and P values < 0.05 were considered to be statistically significant. Statistical analysis was conducted using the SAS, version 9.4 (SAS Institute) and R, version 4.0.3 (R Foundation).

## Results

### Baseline characteristics

A total of 1484 patients (mean age, 63.42 ± 11.26 years; 67.99% male) were enrolled and classified in this analysis, containing white-collar(*n* = 369), blue-collar(*n* = 559) and farmer(*n* = 596). Table 1 presents the baseline characteristics of participants according to occupational class. Between three groups, the distribution of some characteristics significantly differed (*P* < 0.001), such as age, sex, education, residence, employment, baseline National Institutes of Health Stroke Scale score, history of hyperlipidemia and history of diabetes mellitus. However, no significant difference was observed in this analysis between groups in terms of current cigarette smoking, current alcohol drinking, time from stroke onset to hospitalization, systolic BP, total cholesterol, family history of stroke and history of infection before onset.

**Table 1:** Characteristics of 1484 ischemic stroke patients in IIPAIS

Characteristics*	Total (1484)	White-collar (369)	Blue-collar (559)	Farmer (556)	*P* value
Demographic					
Age,y	63.42 ± 11.26	64.69 ± 11.91	64.19 ± 10.98	61.81 ± 10.92	<0.001
Men	1009 (67.99)	277 (75.07)	399 (71.38)	333 (59.89)	<0.001
Education					
Illiteracy	115 (7.75)	12 (3.25)	26 (4.65)	77 (13.85)	<0.001
Primary	490 (33.02)	43 (11.65)	127 (22.72)	320 (57.55)	<0.001
Middle	726 (48.92)	199 (53.93)	374 (66.91)	153 (27.52)	<0.001
College	153 (10.31)	115 (31.17)	32 (5.72)	6 (1.08)	<0.001
Current cigarette smoking	592 (39.89)	140 (37.94)	227 (40.61)	225 (40.47)	0.676
Current alcohol drinking	508 (34.23)	135 (36.59)	176 (31.48)	197 (35.43)	0.208
Urban	905 (60.98)	337 (91.33)	504 (90.16)	64 (11.51)	<0.001
Employed	343 (23.11)	120 (32.52)	127 (22.72)	96 (17.27)	<0.001
Clinical features					
Time from stroke onset to hospitalization, h					0.889
0–24	1210 (81.54)	304 (82.38)	454 (81.22)	452 (81.29)	
24–72	274 (18.46)	65 (17.62)	105 (18.78)	104 (18.71)	
Systolic BP (mmHg)	149.29 ± 21.09	148.36 ± 20.16	148.69 ± 21.56	150.52 ± 21.19	0.217
Diastolic BP (mmHg)	88.53 ± 12.59	87.32 ± 12.06	87.84 ± 13.04	90.02 ± 12.35	0.002
Heart rate (bpm)	74.81 ± 10.55	75.31 ± 10.31	74.64 ± 10.92	74.65 ± 10.34	0.579
Baseline NIHSS score	5.00 (3.00–8.00)	5.00 (3.00–8.00)	4.00 (2.00–7.00)	5.00 (3.00–9.50)	<0.001
NIHSS score at discharge	3.00 (1.00–6.00)	3.00 (1.00–5.00)	3.00 (1.00–5.00)	3.00 (2.00–7.00)	0.011
Ischemic stroke subtype					
Thrombotic	688 (54.69)	180 (55.73)	247 (52.55)	261 (56.13)	0.498
Embolic	69 (4.65)	17 (4.61)	27 (4.83)	25 (4.50)	0.018
Lacunar	525 (41.73)	129 (39.94)	201 (42.77)	195 (41.94)	0.725
Serological examination					
FPG (mmol/L)	5.70 (5.05–7.22)	6.00 (5.20–7.50)	5.70 (5.01–7.47)	5.60 (4.98–6.90)	0.001
Total cholesterol (mmol/L)	4.98 (4.24–5.80)	5.00 (4.26–5.62)	4.89 (4.23–5.78)	5.06 (4.24–5.86)	0.473
Triglycerides (mmol/L)	1.47 (1.08–2.09)	1.54 (1.08–2.18)	1.42 (1.03–2.10)	1.47 (1.11–1.96)	0.298
LDL (mmol/L)	3.20 (2.55–3.79)	3.20 (2.61–3.82)	3.20 (2.59–3.79)	3.16 (2.51–3.77)	0.586
HDL (mmol/L)	1.19 (0.98–1.43)	1.15 (0.93–1.36)	1.15 (0.98–1.41)	1.24 (1.04–1.50)	<0.001
Disease history					
Hypertension	901 (60.71)	245 (66.40)	340 (60.82)	316 (56.83)	0.014
Hyperlipidemia	165 (11.12)	63 (17.07)	62 (11.09)	40 (7.19)	<0.001
Coronary heart disease	229 (15.43)	70 (18.97)	98 (17.53)	61 (10.97)	0.001
Diabetes mellitus	292 (19.68)	103 (27.91)	123 (22.00)	66 (11.87)	<0.001
Family history of stroke	365 (24.60)	107 (29.00)	129 (23.08)	129 (23.20)	0.077
Treatment during hospitalization					
Anticoagulants	339 (22.84)	81 (21.95)	126 (22.54)	132 (23.74)	0.799
Antiplatelet agents	1431 (96.43)	356 (96.48)	536 (95.89)	539 (96.94)	0.635
Thrombolysis	59 (3.98)	17 (4.61)	14 (2.50)	28 (5.04)	0.075
Antihypertensive	729 (49.12)	195 (52.85)	253 (45.26)	281 (50.54)	0.054
Glucose-lowering agents	275 (18.53)	92 (24.93)	108 (19.32)	75 (13.49)	<0.001

Abbreviations: *IIPAIS* Infectious Factors, Inflammatory Markers, and Prognosis of Acute Ischemic Stroke; *BP* blood pressure; *NIHSS* National Institute of Health Stroke Scale; *HDL* high-density lipoprotein; *LDL* low-density lipoprotein; *FPG* fasting plasma glucose

* Continuous variables are expressed as mean ± standard deviation or median (interquartile range). Categorical variables are expressed as frequency (%)

### Association between occupation class and ischemic stroke prognosis

**Fig. 1:**
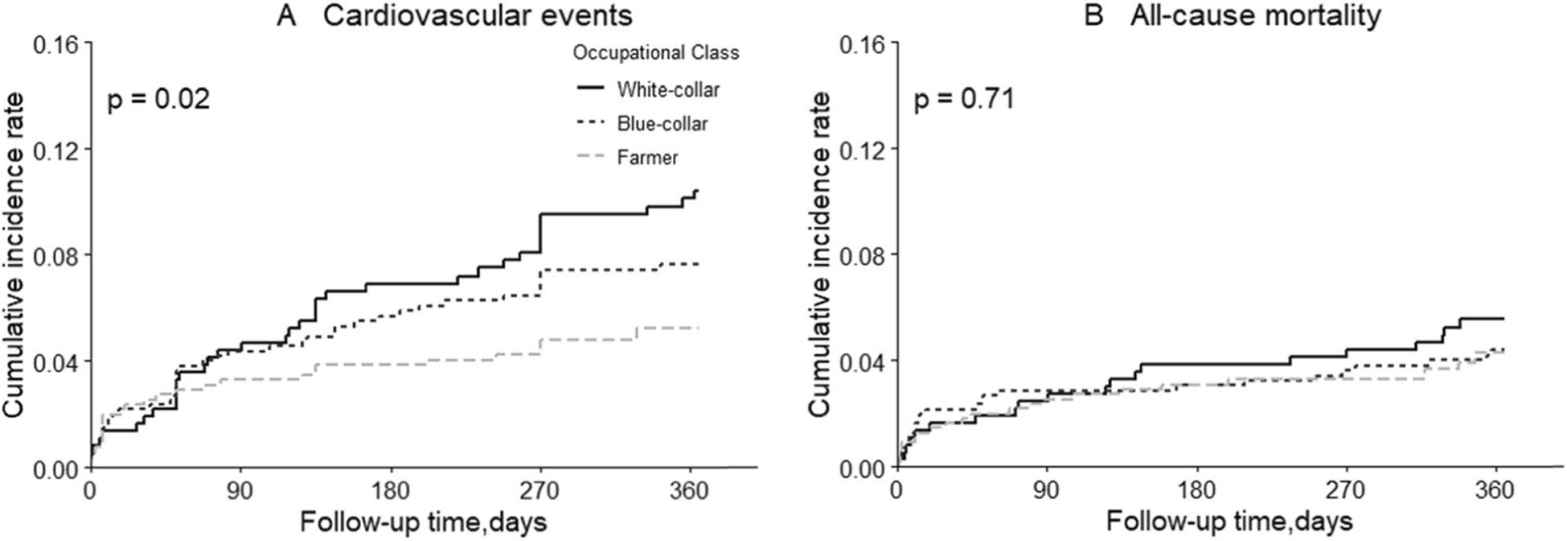
Kaplan–Meier survival curves of cardiovascular events (**A**) and all-cause mortality (**B**) according to occupational class

There were 106 (7.5%) cardiovascular events and 69 (4.9%) all-cause deaths during one year of follow-up (Table 2). From white-collar to blue-collar and farmer group, the incidence densities of cardiovascular events were 113.15 (PYs/1000), 83.47(PYs/1000) and 56.82(PYs/1000), respectively. Generally, Kaplan–Meier plots showed that white-collar workers had highest risk of cardiovascular events after 12-month follow-up (Log-rank *P* = 0.02; Fig. 1).

**Table 2:** Association with occupational class and prognosis during 12-month follow-up

	White-collar (reference)	Blue-collar		Farmer	
		HR(95%CI)	*P* value	HR(95%CI)	*P* value
Cardiovascular events					
Cases,n(%)	37 (10.03)	41 (7.33)		28 (5.04)	
Cases/PYs(/1000)	113.15	83.47		56.82	
Model 1	1.00	0.74 (0.47–1.15)	0.176	0.50 (0.31–0.82)	0.006
Model 2	1.00	0.74 (0.48–1.16)	0.190	0.54 (0.33–0.88)	0.015
Model 3	1.00	0.66 (0.41–1.06)	0.086	0.43 (0.20–0.91)	0.028
All-cause mortality					
Cases,n(%)	20 (5.80)	24 (4.71)		25 (5.03)	
Cases/PYs(/1000)	57.94	46.31		49.06	
Model 1	1.00	0.80 (0.44–1.44)	0.454	0.81 (0.45–1.46)	0.480
Model 2	1.00	0.82 (0.45–1.49)	0.511	1.00 (0.55–1.83)	0.999
Model 3	1.00	0.65 (0.34–1.25)	0.198	0.62 (0.23–1.67)	0.347

Abbreviations: *HR* hazard ratio; *CI* confidence interval; *PY* person year

Model 1: adjusted for age and sex;

Model 2: adjusted for model 1 and further adjusted for current cigarette smoking, current alcohol drinking, time from stroke onset to hospitalization, ischemic stroke subtype, baseline NIHSS score, diastolic blood pressure, high-density lipoprotein cholesterol, fasting plasma glucose, medical history (hypertension, hyperlipidemia, coronary heart disease, diabetes mellitus, family history of stroke), treatment during hospitalization (hypoglycemic, anticoagulants, antiplatelet agents, thrombolysis), NIHSS score at discharge, education, urban and employed

In the unadjusted Cox proportional hazard model (model 1), compared with white-collar workers, farmers had fewer cardiovascular events (HR 0.50, 95% CI 0.31–0.82), while there was no significant difference in blue-collar workers (HR in cardiovascular events 0.74, 95% CI 0.47–1.15) (Table 2). After additional adjustment for current cigarette smoking, current alcohol drinking, time from stroke onset to hospitalization, ischemic stroke subtype, baseline NIHSS score, diastolic blood pressure, high-density lipoprotein cholesterol, fasting plasma glucose, medical history (hypertension, hyperlipidemia, coronary heart disease, diabetes mellitus, family history of stroke), treatment during hospitalization (hypoglycemic, anticoagulants, antiplatelet agents, thrombolysis), NIHSS score at discharge, education, urban and employed (model 3), the adjusted HRs for the blue-collar class and the farmer class were 0.66 (95% CI, 0.41–1.06) and 0.43 (95% CI, 0.20–0.91) for cardiovascular events, compared with the white-collar class, respectively.

### Subgroup analysis

**Fig. 2:**
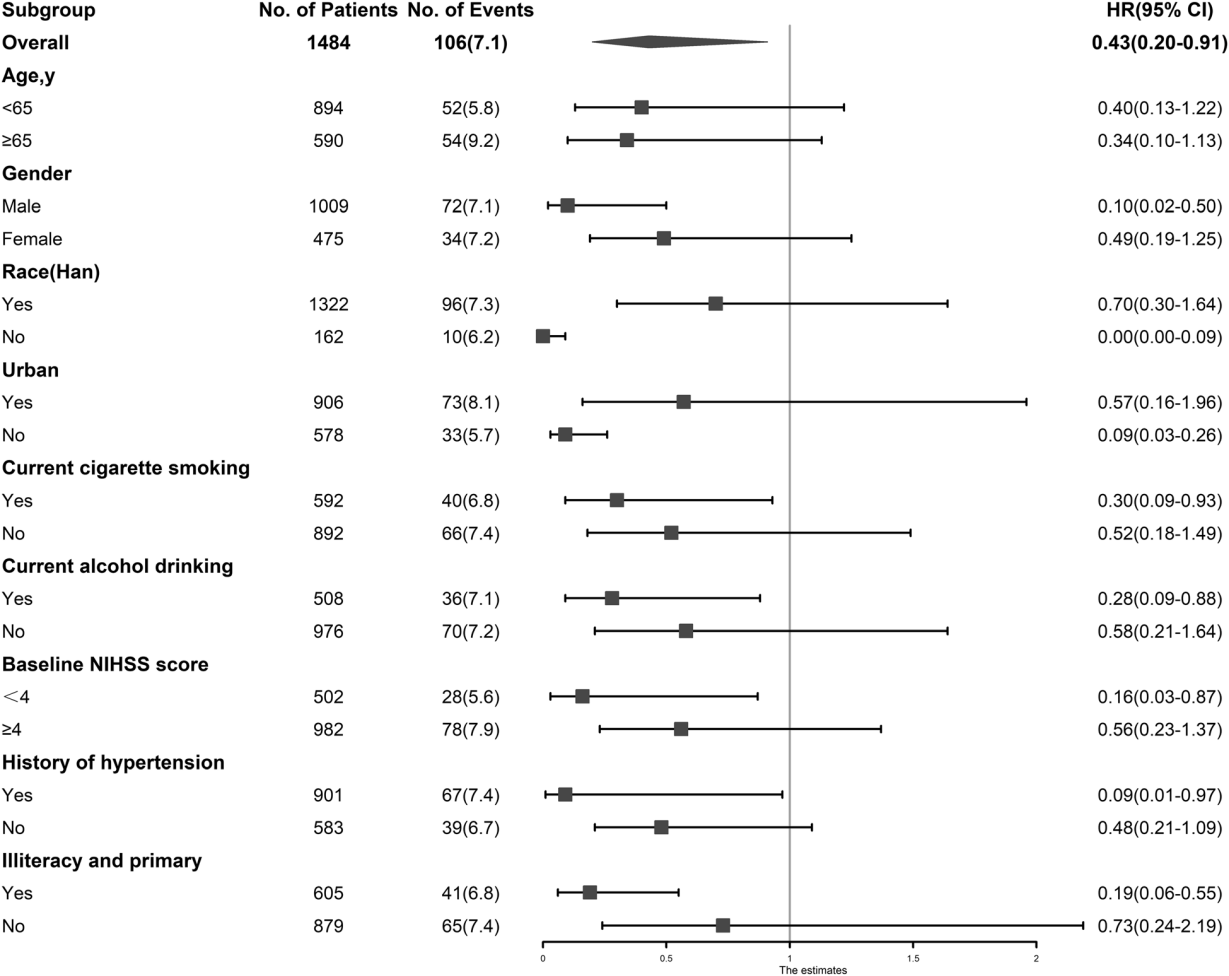
Subgroup analyses of the association between occupation (white-collar and farmer) and cardiovascular events after stroke. Hazard ratios (HRs) were calculated for farmers versus white-collar workers after adjustment for the same variables as model 3 in Table 2, except for the stratified variable. Abbreviations: CI, confidence interval; and NIHSS, National Institutes of Health Stroke Scale. HR, hazard ratio

In the subgroup analysis, the results in age subgroup were consistent. While other covariates, including gender, residence, baseline NIHSS score, current cigarette smoking, current drinking and history of hypertension, to some extent, modified the association between occupational class and cardiovascular events outcome (Fig. 2).

## Discussion

Despite occupational class was considered to be a potential fundamental social factor for cardiovascular disease risk, little was known about the associations between the occupational class and the posttreatment conditions of stroke [11]. In this prospective study, we observed that compared with white-collar patients, patients working as a farmer had a lower risk of cardiovascular events within 12 months after stroke, after adjustment for several traditional stroke risk factors. However, we found no systematic differences in the association between occupational class and all-cause mortality.

Generally, previous evidence on the relationship between occupational class and incidence of stroke is inconsistent. McFadden and colleagues demonstrated that higher social class was associated with decreased incidence of stroke in UK, both in men and women [24]. Moreover, in a matched hospital case-control study in Japan, including 41,038 stroke patients, managers/professionals had lower stroke risk than the blue-collar [25]. Within a population-based stroke registry, low socioeconomic status in any life stage is associated with an increased risk of stroke [26]. While other studies implied that low occupational status had a significantly lower risk of stroke. In a cohort study in Sweden, older men with the lowest occupational status,like unskilled manual, had a significantly lower risk of ischemic stroke [27]. Xu F et found a significantly elevated OR of stroke prevalence in white collar workers compared to blue collar workers, after adjustment for traditional risk factors [22]. However, these studies focused on the incidence of stroke rather than the poor outcomes, and they, to some extent, had insufficient adjustment for confounders.

The occupation groups have relatively distinct social and environmental conditions of work, including occupational physical activities, work schedule demands and work stress, which could partly explain the difference between the occupational groups [28, 29]. The reasons for the difference could be attributed to the difference in work environment, social background, life style and so on. In a large prospective study in China, which enrolled 487,334 study participants, higher level of occupational physical activity was associated with lower risks of major vascular events in adults [30]. Similarly, previous studies found that sitting occupation and sedentary behavior are linked to an increased risk of stroke [31, 32]. In a French population-based cohort, exposure of long working hours for 10 years or more could increase the risk of stroke [33]. Several studies have found that pre-existing poor mental health has a significant impact on long-term stroke outcome [34, 35]. Furthermore, psychological stress at work, which differs in occupation categories, is associated with poor mental health and can increase the risk of cardiovascular disease [36–38] Taken together, these findings are similar to our results that white-collars, who usually work long hours, experience less occupational physical activities, suffer from diabetes mellitus more and are exposed to high job strain with long-time sedentary behavior [39], have more risk factors of cardiovascular events.

With aging population and increasing prevalence of ischemic stroke, China is bearing almost biggest stroke burden around the world. Although low education, income and composite SES was associated with increased risk of stroke mortality both in Chinese and caucasians, the risk of stroke mortality in patients with low education, income, and occupation in Europe was lower than China [14, 40, 41]. This may be due to smaller socioeconomic inequalities and the disparities of work conditions (such as high job strain, insufficient occupational physical activities, work schedule and so on) [25, 30]. Besides, multiple genetic factors and environmental exposure may contribute to this difference [14, 42].

Over the past few decades, China has undergone rapid improvement in economic and society with demographic transitions and lifestyle changes, as well as occupational composition alteration. However, there was recognized inequality between different occupation in China due to disparities in education, rural-urban residence, income, social welfare and healthcare services [40, 43–45]. Therefore, we are supposed to pay more attention to occupational difference in stroke patients. Correspondingly, preventive interventions of health promotion targeting people at high risk occupational groups would be helpful in campaigns to reduce stroke incidence and improve the prognosis. Additionally, occupational therapy, which is referred to the therapeutic use of work, self-care, and playing activities to improve occupational performance and social participation, can be a good choice for those suitable population [46, 47].

The strengths of our study included rigid quality control and relatively comprehensive data collection. Previous studies mainly paid attention to the association between occupational class and stroke incidence [22, 27, 31, 43, 48], while our study was concerned about various short-term ischemic stroke outcomes. Nevertheless, certain limitations should be noted. First, the incidence of cardiovascular events in our study during 12-month follow-up is relatively low [3]. However, in our study, the median NIHSS score was 5 (interquartile range, 3–8) at baseline and 3 (interquartile range, 1–6) at discharge, most patients had a lower NIHSS, which means the majority of participants had mild stroke. In addition, our participants had received good secondary prevention during hospitalization. Therefore, the incidence of cardiovascular events was relatively lower. Second, homemakers and freelance were excluded due to limited numbers and patients included were all from China, thereby affecting external generalizability. Further work is urgently required to incorporate bigger sample size and more occupational classes. Third, residual confounding is inevitable in spite of adjustment for main potential confounders in analysis. For example, we did not collect the data, such as household income, working hours, occupational physical activities and mental health which may influence the difference between occupational groups, especially among those patients in low and middle income countries [30]. Besides, rehabilitation after discharge, an important indicator to assess the outcome of stroke, was not included in this study. Furthermore, to some extent, the data was out-of-date, which makes it cautious to generate the findings to today’s patients. Thus, there is need of further studies with larger size to examine the relationship between more occupational groups and short-term and long-term outcomes of stroke in China.

## Conclusion

In a word, farmers were associated with less risk of cardiovascular events within 12 months after ischemic stroke, compared with white-collar workers. Further prospective studies should be conducted among different occupational populations to improve our findings.

## Data Availability

The datasets used and analyzed during the current study are available from the corresponding author on reasonable request.

## Abbreviations

IIPAIS: Infectious Factors, Inflammatory Markers, and Prognosis of Acute Ischemic Stroke
CIs: Confidence intervals
NIHSS: National Institutes of Health Stroke Scale
TOAST: Trial of Org 10,172 in Acute Stroke Treatment
BP: Blood pressure
SD: standard deviation
IQR: median with interquartile range
MoCA: Montreal Cognitive Assessment
HRs: Hazard ratios
PY: Person year
OR: Odds ratio
UK: United Kingdom
